# Avian influenza H9N2 seroprevalence among pig population and pig farm staff in Shandong, China

**DOI:** 10.1186/s12985-015-0265-9

**Published:** 2015-03-01

**Authors:** Song Li, Yufa Zhou, Yuxin Zhao, Wenbo Li, Wengang Song, Zengmin Miao

**Affiliations:** College of Basic Medicine, Taishan Medical University, Taian, 271000 China; Animal Husbandry Bureau of Daiyue District, Taian, 271000 China; College of Veterinary Medicine, Shanxi Agricultural University, Taigu, 030800 China; College of Life Sciences, Taishan Medical University, Changcheng Road 619, Taian, 271000 China

**Keywords:** Pig population, Pig farm staff, H9N2 Avian influenza virus (AIV), Hemagglutination inhibition (HI), Micro-neutralization (MN)

## Abstract

**Background:**

Shandong province of China has a large number of pig farms with the semi-enclosed houses, allowing crowds of wild birds to seek food in the pig houses. As the carriers of avian influenza virus (AIV), these wild birds can easily pass the viruses to the pigs and even the occupational swine-exposed workers. However, thus far, serological investigation concerning H9N2 AIV in pig population and pig farm staff in Shandong is sparse.

**Methods:**

To better understand the prevalence of H9N2 AIV in pig population and pig farm staff in Shandong, the serum samples of pigs and occupational pig-exposed workers were collected and tested for the antibodies for H9N2 AIV by both hemagglutination inhibition (HI) and micro-neutralization (MN) assays.

**Results:**

When using the antibody titers ≥40 as cut-off value, 106 (HI: 106/2176, 4.87%) and 84 (MN: 84/2176, 3.86%) serum samples of pigs were tested positive, respectively; 6 (HI: 6/287, 2.09%) and 4 (MN: 4/287, 1.39%) serum samples of the pig farm staff were positive, respectively; however, serum samples from the control humans were tested negative in both HI and MN assays.

**Conclusions:**

These findings revealed that there were H9N2 AIV infections in pig population and pig farm staff in Shandong, China. Therefore, it is of utmost importance to conduct the long-term surveillance of AIV in pig population and the pig farm staff.

## Background

Influenza virus, belonging to the orthomyxoviridae family, is a single-stranded, negative-sense, segmented RNA virus. Based on the different antigenicities of nucleoproteins and membrane proteins, these viruses can be categorized into A, B, and C types, among which type A viruses can be further sub-categorized based on their variant surface glycoproteins [hemagglutinin (HA) and neuraminidase (NA)]. Up to date, HA has 18 sub-types and NA has 11 sub-types, and different combinations of HA and NA can generate many viral sub-types [1,2]. Type A influenza viruses have a wide range of biological hosts including human, various avian species, canine, feline, swine, and even marine mammals. According to the different host preferences, influenza viruses can be further categorized into human influenza, avian influenza, and swine influenza viruses [3].

In general, the range of host species infected by the influenza virus is specific and depends on the affinity of receptor binding sites in the HA sequence (mainly on the 226^th^ amino acid) to the surface receptors on the host cells [4]. Two receptor types of influenza virus have been discovered, which are sialic acid α-2,6-galactosidase (SAα-2,6-Gal) and sialic acid α-2,3-galactosidase (SAα-2,3-Gal), respectively. Influenza viruses are selective for the receptor they recognize and subsequently bind. For example, the 226^th^ amino acid in the HA of human influenza virus is leucine, which can bind specifically to SAα-2,6-Gal on the surface of human epithelial cells; the 226^th^ amino acid in the HA of avian influenza virus is glutamine, which specifically binds to SAα-2,3-Gal on the surface of avian epithelial cells; and the 226^th^ amino acid in the HA of swine influenza virus is methionine, which has equal binding affinity for SAα-2,6-Gal and SAα-2,3-Gal on the surface of swine epithelial cells [5-9]. Therefore, pigs are the common susceptible host of avian, swine, and human influenza viruses, and are the living vectors of different influenza viruses for gene reassortment, resulting in the generation of new influenza virus sub-types [10,11]. The novel H1N1 influenza virus in 2009 is a triple-reassortant, containing fragments of avian, human, and swine influenza viruses [12]. Therefore, timely surveillance of AIV infection in pig population is of importance.

Following the first discovery of H9N2 AIV in Guangdong province of mainland China in 1994, the virus spread rapidly in China and so far has become the most prevalent influenza virus sub-type in poultry [13]. Shandong province has a large number of pig farms with semi-enclosed houses, which allows crowds of wild bird to seek food in the pig farms. As the carriers of H9N2 AIV, these wild birds can easily pass the virus to pig population and even the pig farm staff, providing the opportunity for the reassortment of different sub-types of influenza viruses [14,15]. However, thus far, the serological investigation on H9N2 AIV in pig population and pig farm staff in Shandong is sparse. Therefore, the study was performed to further understand the prevalence of H9N2 AIV infections in pig population and pig farm staff in Shandong province, China.

## Materials and methods

### Sample collection

**Figure 1:**
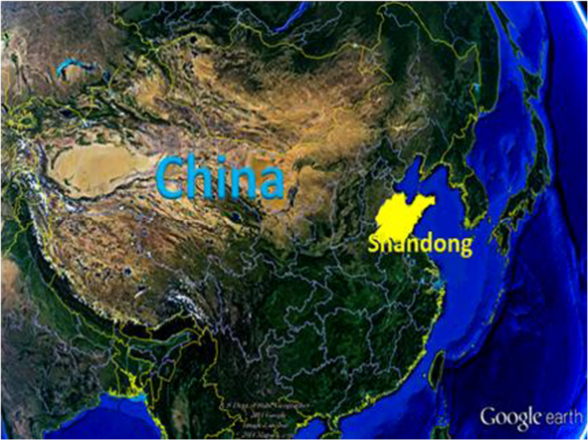
**Location of Shandong province (highlighted) in China.**

**Figure 2:**
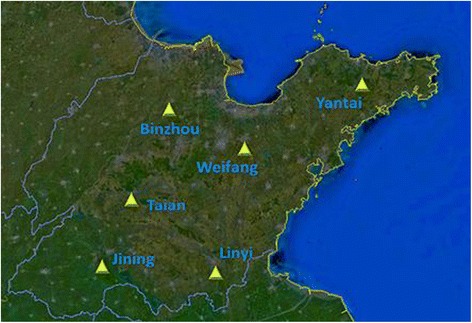
**Sample collection sites in Shandong, China.**

Blood samples were collected from 50 pig fattening farms in 6 regions in Shandong province between May 2013 and April 2014 (Figures 1 and 2). All of these pig farms used the all-in-all-out breeding mode with semi-enclosed pig houses, and the breeding scales were from 500 to 1000 pigs. Since no separation nets were used in the pig farms, many wild birds frequently sought food in the pig houses and thus had chances to come into closer contact with the pigs, and even the staff. After obtaining the consent of the farmers, healthy fattening pigs weighing 30–50 kg that were not vaccinated with any influenza were randomly selected, and 30–50 blood samples were collected from each pig farm.

After obtaining written informed consent, adults aged ≥18 years were enrolled in the study. A random-number-generated sample was collected among pig farm staff working at 50 pig farms in Shandong, enrolling 287 participants. A total of 100 control serum samples were also collected from the healthy control humans in Shandong. All participants were interviewed with a standard questionnaire on demographic characteristics, the history of poultry contact, and the history of vaccination of influenza. Peripheral blood samples (5 ml each) were obtained from all the participants for the determination of the antibody to H9N2 AIV. The serum samples were separated and stored at −20°C until use.

### Ethics statement

This study was approved by the ethics committee of the Shandong Center for Disease Control and Prevention.

### Viruses and antigens

**Figure 3:**
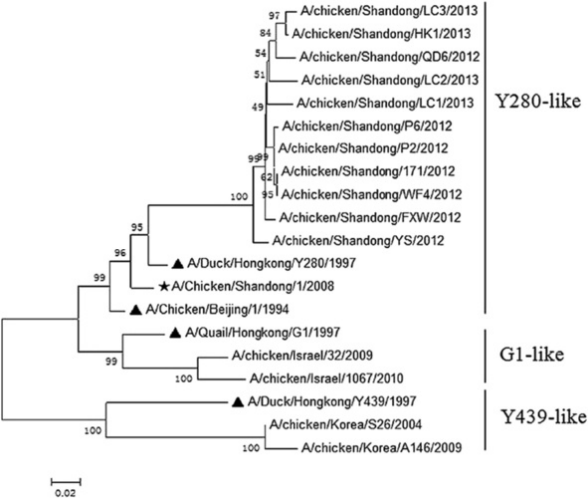
**Phylogenetic tree of HA genes of H9N2 avian influenza viruses isolated in Shandong from 2012 to 2013.** Notes: The reference viruses are marked with a black triangle, and the used virus in this study (A/chicken/Shandongdong/1/2008) is marked with a black star.

Phylogenetic analysis of the HA genes from H9N2 AIV from 2012 to 2013 in Shandong found that the epidemic H9N2 AIV in this region belongs to the Y280-like lineage (Figure 3). Therefore, Shandong’s local viral isolate (A/chicken/Shandong/1/2008) was used as the detection antigen.

### Preparation of collected serum samples

Before the HI and MN assays were performed, receptor-destroying enzyme (RDE) was added at 3 times volume of the collected serum samples. The mixture was placed in a 37°C water bath overnight to remove nonspecific inhibitor present in the serum samples, and was then placed in a water bath at 56°C for 30 min to inactivate redundant RDE. Chicken erythrocytes (20%) in 1/5 volume of the serum sample was added to remove nonspecific HAs in serum samples and the mixture was placed in a water bath at 4°C for 30 min, and was then centrifuged at 2,000 rpm for 10 min to remove erythrocytes. Physiological saline was added to the processed serum sample at a dilution ratio of 1:10. The sample was numbered and preserved at −20°C until further use.

### Hemagglutination inhibition (HI) assay

A prepared serum sample (25 μL) was mixed with an equal amount of physiological saline (0.9%), and was then applied to a 96-well plate for serial dilution. H9N2 AIV antigen (25 μL of 4-unit stock) was added to each well after dilution, mixed by mild vibration and then cultivated at room temperature for 30 min; 50 μL of 0.5% chicken erythrocytes was added to each well, mixed and then cultured at room temperature for 30 min. The HI titers were expressed as the highest serum dilution ratio required for complete inhibition of erythrocyte hemagglutination [16-18].

### Micro-neutralization (MN) assay

A prepared HI positive serum sample (50 μL) was diluted with an equal amount of physiological saline in a 96-well plate for serial dilution, and then an equal amount (50 μL) of diluted virus at a concentration of 1 × 10^2^ TCID50/mL was added and mixed by gentle flicking. The virus and serum mixture was cultured for 1 h in a 5% CO_2_ incubator at 37°C and then 100 μL of virus and serum solution mixture was inoculated onto a monolayer of Madin-Darby canine kidney epithelial (MDCK) cells in a 96-well cell culture plate, which was then incubated in a 5% CO_2_ incubator at 37°C. The inoculum was aspirated after 1 h and 100 μL of minimal essential medium (MEM) supplemented with 10% bovine serum was added to each well. The plate was then incubated in a 5% CO_2_ incubator at 35°C for 2 days. The cytopathic effects were observed every day using an inverted microscope. MN titers were expressed as the serum dilution ratio for inhibition of the cytopathic effect in 50% MDCK cells [17,18].

### Statistical analysis

The chi-square test was used to evaluate the difference in positive samples between pig farm staff and university students and the difference in positive pig samples between different pig farms. All statistical analysis was conducted using StataCorp. 2011. Stata Statistical Software: Release 12. College Station, TX: StataCorp LP. If the *p*-value is under 0.05, results are considered statistically significant.

## Results

### Analysis of pig serum samples

Serum samples from 2,176 pigs were successfully collected from 50 pig fattening farms in 6 regions, among which there were 420 pigs from Yantai, 510 from Weifang, 326 from Binzhou, 320 from Taian, 200 from Jining, and 400 from Linyi (Table 1). When HI and MN antibody titers ≥40 was set cut-off value, 106 samples showed positive in the HI assay (4.87%, 106/2176), and 84 samples showed positive in the MN assay (3.86%, 84/2176). When HI and MN antibody titers ≥80 was set as the positive result, 48 samples showed positive in the HI assay (2.21%, 48/2176), and 32 samples showed positive in the MN assay (1.47%, 32/2176). When using ≥160 as the cut-off value, no positive samples were observed. When HI and MN titers ≥40 was set as the cut-off value, these 50 pig-fattening farms did not experience a statistically significant outbreak in terms of the H9N2 AIV infection rate (*P* = 0.647).

**Table 1:** **HI and MN results of pig serum samples**

**Locations (number of samples)**	**Antibody titers (%)**			
	**≥40**		**≥80**	
	**HI**	**MN**	**HI**	**MN**
Yantai (420)	19 (4.52)	15 (3.57)	9 (2.14)	6 (1.43)
Weifang (510)	27 (5.29)	22 (4.31)	12 (2.35)	8 (1.57)
Binzhou (326)	14 (4.29)	11 (3.37)	7 (2.15)	5 (1.53)
Taian (320)	16 (5.00)	12 (3.75)	7 (2.19)	5 (1.56)
Jining (200)	9 (4.50)	7 (3.50)	4 (2.00)	3 (1.50)
Linyi (400)	21 (5.25)	17 (4.25)	9 (2.25)	5 (1.25)
Total (2176)	106 (4.87)	84 (3.86)	48 (2.21)	32 (1.47)

### Analysis of human serum samples

Human serum samples (387) were collected in this study from 2 test groups: 100 samples were from the healthy subjects as a control group, and 287 samples were from humans working at 50 pig farms as the experimental group. None of the subjects that provided serum samples had received any kind of influenza vaccination or had experienced any influenza-like symptoms within 6 months before providing serum samples (Table 2).

**Table 2:** **Demographic breakdown of sero-epidemiological survey by locations**

**Locations**	**No.**	**No. demographic description**			
		**Median of age (range)**	**No. of female (%)**	**Occupation**	**Average occupational year**
Yantai	55	49 (46–60)	18 (32.7)	Swine farm employee	5
Weifang	70	50 (45–58)	20 (28.6)	Swine farm employee	4
Binzhou	42	46 (42–55)	10 (23.8)	Swine farm employee	8
Taian	40	52 (49–61)	12 (30.0)	Swine farm employee	3
Jining	38	46 (42–57)	10 (26.3)	Swine farm employee	5
Linyi	42	45 (42–58)	8 (19.0)	Swine farm employee	5
Taian	100	21 (19–23)	50 (50.0)	University students	3

Survey site where the sero-epidemiological survey was conducted.

Sampling site where the control serum samples was collected.

When HI and MN antibody titers ≥40 was used as cut-off value, serum samples collected from the subjects in the control group showed negativity in both assays; the results of the pig farm staff showed that 6 samples showed positive in the HI assay (2.09%, 6/287) and 4 samples showed positive in the MN assay (1.39%, 4/287). When ≥80 was set as cut-off value, 4 samples of the pig farm staff showed positive in the HI assay (1.39%, 4/287), and 2 samples showed positive in the MN assay (0.69%, 2/287) (Tables 3 and 4). When HI and MN antibody titers ≥40 was set as the positive result, the difference in positive samples between swine farm staff and the control subjects was statistically significant (2.09% vs. 0%, *P* = 0.027).

**Table 3:** **HI and MN results of human serum samples**

**Humans**	**Antibody titers**			
	**≥40**		**≥80**	
	**HI**	**MN**	**HI**	**MN**
Swine farm employees (n = 287)	6	4	4	2
Positive rate (%)	2.09	1.39	1.39	0.69

**Table 4:** **Six swine farm workers of with the positive results of HI and MN**

**Locations**	**Age (years)**	**Gender**	**Results**	
			**HI**	**MN**
Weifang	56	Female	160	80
Weifang	52	Female	40	--
Taian	46	Male	40	--
Linyi	49	Female	160	160
Jining	45	Male	80	40
Binzhou	50	Female	80	40

## Discussion

Since H9N2 AIV was first isolated from the diseased chickens in mainland China in 1994 [19], the host range of the virus has become very wide. For example, Researchers have successfully isolated this virus from pigs and found that it can also infect humans [20,21]. Pigs are considered as the “mixer” and “amplifier” for the recombination of human, swine and avian influenza viruses, providing the conditions for gene recombination of influenza viruses [22]. With the increasing prevalence of avian influenza, it is of utmost importance to test pigs and pig farm staff for H9N2 AIV infections.

To exclude the cross-reaction caused by infection with other influenza viruses, we defined the positive antibody titers to H9N2 AIV as ≥40 and ≥80, which not only can met the World Health Organization (WHO) diagnostic criterion, most importantly, but can also avoid the cross-reaction to some extent that may result from antibodies to H1N1 and H3N2 influenza viruses [18]. Previous studies also proved that when the cut-off value of HI antibody titres to H9N2 AIV was ≥40, the cross-reaction with other influenza viruses was not evident [23,24]. Additionally, to further reduce the cross-reaction with other seasonal influenza viruses, the selected pig population and the humans in this study were not vaccinated against any influenza.

Based on the cut-off value of HI and MN antibody titers as ≥40, the seroprevalence of pigs in Shandong was 4.87% and 3.86%, respectively; and the serum samples of 6 pig farm staff were positive; but no positive serum samples were detected in the control humans. The results showed that the H9N2 AIV infections existed in pig population and the pig farm staff in Shandong region, which is in agreement with the results of the previous studies in China [25,26].

The results of this study suggested that there was H9N2 AIV infection among pigs and pig farm staff in Shandong. Although no clinical cases of H9N2 infection were found during the investigation, the possibilities of potential or subclinical infections cannot be ruled out. The lack of necessary separation nets caused many wild birds to seek food in pig houses, which may be one of the most important routes leading to H9N2 AIV infection in pig population and the occupational swine-exposed workers.

## Conclusions

This study revealed that there were H9N2 AIV infections in pig population and the pig farm staff in Shandong region. Therefore, it is of utmost importance to carry out the long-term surveillance of AIV in pig population and the occupational swine-exposed humans.
